# The trajectory of sleep after critical illness: a 24-month follow-up study

**DOI:** 10.1186/s13613-025-01449-9

**Published:** 2025-02-28

**Authors:** Mario Henríquez-Beltrán, Iván D. Benítez, Rafaela Vaca, Sally Santisteve, Maria Aguilà, Anna Vila, Olga Minguez, Carlos Rodríguez-Muñoz, Anna Galán-González, Sulamita Carvalho-Brugger, Paula González, Paula Rodríguez, Jesús Caballero, Carme Barberà, Gerard Torres, Gonzalo Labarca, Mar Malla-Banyeres, Anna Moncusí-Moix, Antoni Torres, David de Gonzalo-Calvo, Ferran Barbé, Jessica González, Adriano D. S. Targa

**Affiliations:** 1 Translational Research in Respiratory Medicine, Hospital Universitari Arnau de Vilanova-Santa Maria, Biomedical Research Institute of Lleida (IRBLleida), Rovira Roure, 80, 25198 Lleida, Spain; 2 CIBER of Respiratory Diseases (CIBERES), Institute of Health Carlos III, Madrid, Spain; 3 Núcleo de Investigación en Ciencias de la Salud, Universidad Adventista de Chile, Chillán, Chile; 4 Intensive Care Department, Hospital Universitari Arnau de Vilanova, Lleida, Spain; 5 Intensive Care Department, Hospital Universitari Santa Maria, Lleida, Spain; 6 Department of Respiratory Diseases, School of Medicine, Pontificia Universidad Católica de Chile, Santiago, Chile; 7 Division of Pulmonary and Critical Care Medicine, Beth Israel Deaconess Medical Center and Harvard Medical School, Boston, USA; 8 Servei de Pneumologia, Hospital Clinic, Universitat de Barcelona, IDIBAPS, Barcelona, Spain

**Keywords:** Sleep, Mental health, Critical survivors, Pittsburgh sleep quality index, SARS-CoV-2

## Abstract

**Background:**

Survivors of critical illness endure long-lasting physical and mental challenges. Despite the persistence of poor sleep quality in a considerable proportion of patients at the 12-month follow-up, studies with assessments exceeding this period are limited. We aimed to investigate the trajectory of sleep over the 24 months following critical illness.

**Methods:**

Observational, prospective study. Patients diagnosed with SARS-CoV-2 infection were recruited during the intensive care unit stay. Evaluations of sleep (Pittsburgh Sleep Quality Index [PSQI]), mental health (Hospital Anxiety and Depression Scale [HADS]), quality of life (12-item Short Form Survey [SF-12]), and other factors were performed in the short-term, and at 12 and 24 months after hospital discharge. Good sleep quality was defined as a PSQI score of ≤ 5. Minimal clinically important improvement (MCII) was defined as a decrease of ≥ 4 points in the PSQI score between the short-term assessment and the 24-month follow-up.

**Results:**

The cohort included 196 patients (69.9% males), with a median [p_25_;p_75_] age of 62.0 [53.0;67.2] years. The global population showed a mean (95% CI) change of − 0.91 ( − 1.50 to − 0.31) points in the PSQI score from the short-term assessment to the 24-month follow-up. Based on PSQI score trajectories, three distinct groups of patients were identified: (i) the *healthy* group, consisting of patients with good sleep quality in the short-term that was maintained throughout the follow-up period; (ii) the *MCII* group, consisting of patients with poor sleep quality in the short-term, but with improvement over time, ultimately reaching levels comparable to the healthy group; (iii) the *non-MCII* group, consisting of those with consistently poor sleep quality across the entire follow-up. Further analyses revealed that PSQI score trajectories were closely aligned with those of the HADS and SF-12 mental scores.

**Conclusions:**

Our findings reveal that a subset of critical illness survivors requires up to 24 months after the acute phase to fully restore their sleep quality, while a significant proportion does not experience a clinically significant improvement in sleep quality over this period. These distinct sleep trajectories are strongly correlated with mental health status, highlighting the importance of addressing sleep alongside mental health within the framework of post-intensive care syndrome.

**Supplementary Information:**

The online version contains supplementary material available at 10.1186/s13613-025-01449-9.

## Background

Survivors of life-threatening illness often experience a myriad of new or exacerbated conditions that may persist for months or even years after the acute phase of the disease [1]. These include physical symptoms such as neuromuscular weakness and reduced autonomy in daily living activities [2, 3], as well as mental symptoms such as anxiety [4], depression [5], post-traumatic stress disorder [6], and neurocognitive alterations that lead to difficulties in thinking, remembering, or concentrating [3].

Sleep plays a crucial role in various bodily functions, including the maintenance of quality of life [7] and immune function [8]. Poor sleep quality is associated with numerous adverse outcomes both in the short [9] and long term [10]. Thus, preserving good sleep health is essential for the recovery of critically ill survivors. Nevertheless, previous studies have reported significant sleep alterations among these patients, both during the acute phase of the disease [11] and after hospital discharge [12–14]. The unprecedented context caused by the SARS-CoV-2 pandemic has further highlighted the extent of sleep disruption in critical illness [15, 16], with up to 60.5% of critical COVID-19 patients reporting poor sleep quality three months after hospital discharge [17], a prevalence that remains high in mid-term [18] and long-term [19] assessments.

Sleep is also closely linked to mental well-being [20], which is essential for the recovery of critically ill patients. Studies have shown significant correlations between sleep and mental health at various stages following hospital discharge, suggesting a bidirectional relationship [21]. However, it is only in recent years that sleep disturbances have been recognized alongside mental health symptoms, such as anxiety and depression, as integral components of post-intensive care syndrome.

In light of this, the aim of this study was to investigate the trajectory of sleep over the 24 months following critical illness. We sought to identify distinct trajectories based on the Pittsburgh sleep quality index (PSQI) scores along the follow-up period. Furthermore, we explored the associations between these and the trajectory of relevant factors for an integrated recovery of critical survivors such as mental health, quality of life, and respiratory function.

## Methods

### Study population

The inclusion criteria comprised: (i) aging 18 years or more, (ii) admission to the intensive care unit (ICU) stay due to SARS-CoV-2 infection between March 2020 and October 2021. The exclusion criteria included: (i) palliative care, (ii) mental and/or physical disabilities after hospital discharge that could prevent the accomplishment of the proposed evaluations, (iii) absence at the follow-up appointments. This study was approved by the Medical Ethics Committee of the Hospital Universitari Arnau de Vilanova (CEIC-2510; ‘The impact of COVID-19 and its context on sleep and circadian rhythms’; June 22, 2021) and conducted according to the principles outlined by the Declaration of Helsinki. Informed consent was acquired for all patients.

### Study design

Prospective, observational, single-center study. Patients were recruited during the ICU stay at the Hospital Universitari Arnau de Vilanova-Santa Maria (Lleida, Spain). Medical appointments occurred at the short-term (median [p_25_;p_75_] elapsed time of 3.8 [3.2;4.8] months), one year (12.4 [12.0;13.1] months) and two years (24.6 [24.0;25.4] months) after hospital discharge. In each visit, subjective assessments of sleep, mental health, and quality of life were performed as well as objective evaluations of respiratory function and aerobic capacity.

### Data obtained during the ICU stay

Sociodemographic and anthropometric information such as age, sex, body mass index (BMI), habits, and comorbidities were obtained through the medical records. Figures related to arterial blood gases (pH, partial pressure of arterial oxygen [PaO_2_], partial pressure of carbon dioxide [PaCO_2_], fractional inspired oxygen [FiO_2_], oxygen saturation [SaO_2_] and hydrogen carbonate [HCO_3_^−^]), and clinical requirements (invasive mechanical ventilation [IMV], non-IMV [NIMV], prone position, pharmacological treatment) were also collected. The APACHE-II (Acute Physiology and Chronic Health disease Classification System II) score was determined based on previous reports [22].

### Data obtained during the follow-up period

#### Pittsburgh sleep quality index (PSQI)

Sleep quality was assessed through the PSQI. This questionnaire is composed of 19 questions representing one of the seven components of sleep quality: subjective sleep quality, sleep latency, sleep duration, sleep efficiency, sleep disturbance, sleep medication intake, and daytime dysfunction. Each component score is rated on a 3-point scale, leading to a sum of up to 21 points. A PSQI score > 5 indicates poor sleep quality whereas a PSQI score ≤ 5 indicates good sleep quality[23, 24].

#### Epworth sleepiness scale (ESS)

Excessive daytime somnolence was assessed through the ESS. This questionnaire is composed of eight questions to assess the chance of falling asleep during different daily situations. Each question is rated on a 3-point scale, in which 0 represents no chance of occurrence, and 3 indicates a high chance of occurrence. The overall score ranges from 0 to 24 points. The total score was categorized as daytime sleepiness (> 10) or no daytime sleepiness (≤ 10) [25, 26].

#### Satisfaction alertness timing efficiency duration (SATED) questionnaire

Sleep was further assessed by the SATED questionnaire. This questionnaire is composed of five questions representing one of the following sleep-related dimensions each: subjective satisfaction, alertness during waking hours, appropriate timing, efficiency, and duration. Each question is rated on a 2-point scale, leading to a sum of up to 10 points. Higher scores indicate better sleep health [27].

#### Hospital anxiety and depression scale (HADS)

The HADS was used to assess signs of anxiety and depression. This questionnaire consists of a 7-item anxiety subscale and a 7-item depression subscale. Each item is rated on a 3-point scale, leading to a sum of up to 21 points. A score > 8 indicates possible anxiety or depression whereas a score ≤ 8 indicates the opposite[28, 29].

#### 12-item short form survey (SF-12)

The SF-12 was used to evaluate the quality of life. The SF-12 is composed of 12 questions comprising the following domains: general health, physical functioning, role-physical, bodily pain, vitality, social functioning, role-emotional, and mental health. A SF-12 score > 50 indicates a good health-related quality of life whereas a SF-12 score < 50 indicates a poor health-related quality of life [30, 31].

#### Spirometry and computed tomography (CT)

Airway function was measured and represented as previously described[32]. The diffusing lung capacity for carbon monoxide (DLCO) was the variable used to represent the respiratory function. CT of the chest was performed to evaluate the severity of lung affectation. To quantify this, we calculated the total severity score.

#### 6-min walking test (6MWT)

The 6MWT was performed to evaluate aerobic capacity[33]. The traveled distance was compared with reference values[34]. Accordingly, the predicted 6-min walked distance (6MWD) was calculated based on the following equations: for men, predicted 6MWD = (7.57 × height)—(5.02 × age)—(1.76 × weight)—309 m; for women, predicted 6MWD = (2.11 × height)—(5.78 × age)—(2.29 × weight) + 667 m. Percent predicted 6MWD (PP-6MWD) was calculated using the formula: PP-6MWD = 6MWD/Predicted 6MWD × 100.

### Trajectories of sleep and study groups

The distinct sleep trajectories were identified based on the PSQI score in the first visit (short-term) and the presence of a minimal clinically important improvement (MCII) in the PSQI score over the follow-up period. MCII was defined as a decrease of ≥ 4 points in the PSQI score between the short-term assessment and the 24-month follow-up [35, 36]. Based on these criteria, participants were categorized into three groups: (i) the *healthy* group, consisting of individuals with good sleep quality (PSQI score ≤ 5) in the short-term that was maintained throughout the follow-up period; (ii) the *MCII* group, comprising individuals with poor sleep quality (PSQI > 5) in the short-term who showed a significant improvement, defined as a decrease of ≥ 4 points in the PSQI score during the follow-up period; (iii) the *Non-MCII* group, including individuals with poor sleep quality (PSQI > 5) in the short-term who did not show a significant improvement, exhibiting a decrease of < 4 points in the PSQI over the 24-month follow-up.

### Statistical analysis

Descriptive statistics were used to summarize the baseline characteristics (sociodemographic and anthropometric data, habits, comorbidities, and information related to hospitalization and ICU stay). Medians [p_25_;p_75_] were estimated for quantitative variables while frequencies (percentages) were used for categorical data.

Comparisons of baseline characteristics between groups were conducted using the chi-square test or Fisher’s exact test for qualitative variables, with Fisher’s exact test applied when expected frequencies were less than 5. For quantitative variables, the Wilcoxon signed-rank test or Kruskal–Wallis test was employed.

To evaluate the trajectory of PSQI, SATED, and ESS during the follow-up period for the global population, we used a linear mixed-effect model, with time introduced as fixed effect and patient as random effect. The trajectories of PSQI, SATED, ESS, HADS (anxiety and depression scores), SF-12 (physical and mental domains), 6MWT (PP-6MWD and mean oxygen saturation [SpO_2_]), spirometry (DLCO), and CT of the chest (total severity score), stratified by study groups, were assessed using a linear mixed-effect model. This model included time, study groups, and the time-group interaction as fixed effects, and patients as random effect. Differences between the study groups at each time-point were assessed considering the interaction between group and time (with time included as a fixed effect).

An additional analysis was conducted to evaluate the trajectory of PSQI over continuous time, stratified by study groups. This analysis employed a multivariate generalized additive mixed model (GAM). Time, study groups, and time-group interaction were included as fixed effects and patients as a random effect. The GAM utilized a simple factor smooth interaction (time vs. group) with a thin plate regression spline.

The p-value threshold defining statistical significance was set at less than 0.05. All statistical analyses were performed using R software, Version 4.0.2 (R Core Team; Vienna, Austria).

## Results

### Baseline characteristics

The study enrolled 332 patients during their ICU stay between March 2020 and October 2021 (Additional file 1: Table S1). The analysis included 196 patients who completed the two-year follow-up. The majority were males (69.9%), with a median [p_25_;p_75_] age of 62.0 [53.0;67.2] years (Table 1). The most prevalent comorbidities were obesity (47.4%), hypertension (45.9%), and diabetes mellitus (21.9%). The median length of hospitalization and ICU stay were 22.0 [14.0;36.2] and 13.0 [7.00;22.0] days, respectively, during which 55.1% of the patients required ventilatory support with IMV.

**Table 1:** Baseline characteristics of the cohort

	Global n = 196
	n (%) or median [p_25_;p_75_]
Sociodemographic and anthropometric data	
Sex, male	137 (69.9%)
Age, years	62.0 [53.0;67.2]
Body mass index, kg·m^2^	29.8 [26.7;33.6]
Habits	
Tobacco	
Former use	91 (48.4%)
Never used	92 (48.9%)
Current use	5 (2.66%)
Chronic alcohol	
Former consumption	2 (1.06%)
Never consumed	180 (95.2%)
Current consumption	7 (3.70%)
Comorbidities	
Obesity	93 (47.4%)
Hypertension	90 (45.9%)
Diabetes mellitus type 2	43 (21.9%)
Asthma	13 (6.63%)
Chronic obstructive pulmonary disease	8 (4.08%)
Hospitalization	
Duration, days	22.0 [14.0;36.2]
Before ICU admission, days	1.00 [0.00;2.00]
After ICU discharge, days	8.00 [5.00;12.0]
ICU stay	
Duration, days	13.0 [7.00;22.0]
APACHE-II score	11.0 [9.00;13.0]
Arterial blood-related information	
pH	7.45 [7.40;7.48]
Partial pressure of oxygen (PaO_2_), mmHg	70.0 [56.0;91.0]
Partial pressure of carbon dioxide (PaCO_2_), mmHg	36.0 [32.2;41.0]
Fractional inspired oxygen (FiO_2_), %	65.0 [50.0;84.5]
PaO_2_ to FiO_2_ ratio	129 [90.1;172]
Oxygen saturation (SaO_2_), %	95.0 [91.3;96.9]
Hydrogen carbonate (HCO_3_^−^), mmol/L	24.7 [21.8;27.4]
Procedures	
Invasive mechanical ventilation	108 (55.1%)
Duration, days	13.0 [8.00;25.0]
Non-invasive mechanical ventilation	147 (75.4%)
Duration, days	3.00 [1.00;5.00]
Prone position	111 (56.9%)
Duration, hours	41.0 [24.0;78.8]
Pharmacotherapy	
Corticosteroids	180 (91.8%)
Antibiotics	157 (80.1%)
Tocilizumab	135 (68.9%)
Hydroxychloroquine	48 (24.5%)
Remdesivir	25 (12.8%)

APACHE-II, acute physiology and chronic health evaluation; FiO_2_, fractional inspired oxygen; ICU, intensive care unit; n, number; p, percentile; PaO_2_, partial pressure of oxygen. Missings: tobacco, 8; chronic alcohol abuse, 7

### Trajectory of sleep along the 24-month follow-up

The mean change (95% CI) in the PSQI score was − 0.75 ( + 1.38 to − 0.13) between the short-term and 12-month visits, and − 0.91 ( − 1.50 to − 0.31) between the short-term and 24-month follow-ups (Table 2) (Additional file 1: Figures S1 and S2). No significant difference between the 12-month and 24-month visits was detected. The scores of specific components of the PSQI (subdomains representing sleep disturbances and daytime dysfunction), SATED questionnaire, and ESS presented a similar pattern, with improvement noted between the short-term and subsequent visits, but no significant change between the 12-month and 24-month follow-ups.

**Table 2:** Trajectory of sleep along the 24-month follow-up

	Short-term	12-month	24-month	12-month vs. Short-term		24-monthvs.Short-term		24-monthvs.12-month	
	Mean (95%CI)			Mean difference (95%CI)	p-value	Mean difference (95%CI)	p-value	Mean difference (95%CI)	p-value
*Pittsburgh sleep quality index (PSQI)*	6.47 (5.90 to 7.04)	5.72 (5.12 to 6.31)	5.56 (4.99 to 6.13)	− 0.75 ( − 1.38 to − 0.13)	0.013	− 0.91 ( − 1.50 to − 0.31)	0.001	− 0.15 ( − 0.78 to 0.47)	0.831
Subjective sleep quality	1.02 (0.93 to 1.11)	1.08 (0.98 to 1.17)	1.07 (0.97 to 1.16)	0.06 ( − 0.06 to 0.17)	0.510	0.05 ( − 0.07 to 0.16)	0.603	− 0.01 ( − 0.13 to 0.11)	0.979
Sleep latency	1.05 (0.90 to 1.20)	0.98 (0.82 to 1.13)	0.89 (0.74 to 1.04)	− 0.07 ( − 0.25 to 0.11)	0.649	− 0.16 ( − 0.33 to 0.01)	0.081	− 0.09 ( − 0.27 to 0.09)	0.477
Sleep duration	1.27 (1.12 to 1.42)	1.11 (0.95 to 1.26)	1.18 (1.03 to 1.33)	− 0.16 ( − 0.34 to 0.02)	0.084	− 0.09 ( − 0.26 to 0.08)	0.416	0.07 ( − 0.11 to 0.25)	0.621
Sleep efficiency	0.99 (0.84 to 1.15)	0.88 (0.72 to 1.04)	0.83 (0.67 to 0.98)	− 0.12 ( − 0.33 to 0.09)	0.392	− 0.17 ( − 0.37 to 0.03)	0.118	− 0.05 ( − 0.26 to 0.16)	0.830
Sleep disturbance	0.89 (0.80 to 0.98)	0.77 (0.67 to 0.86)	0.74 (0.65 to 0.83)	− 0.12 ( − 0.25 to 0.01)	0.074	− 0.15 ( − 0.27 to − 0.02)	0.016	− 0.03 ( − 0.16 to 0.11)	0.889
Sleep medication intake	0.73 (0.56 to 0.91)	0.69 (0.51 to 0.87)	0.69 (0.51 to 0.86)	− 0.05 ( − 0.21 to 0.12)	0.797	− 0.05 ( − 0.21 to 0.11)	0.777	0.00 ( − 0.17 to 0.17)	1.000
Daytime dysfunction	0.52 (0.43 to 0.60)	0.23 (0.14 to 0.33)	0.17 (0.08 to 0.26)	− 0.28 ( − 0.43 to − 0.14)	< 0.001	− 0.34 ( − 0.48 to − 0.21)	< 0.001	− 0.06 ( − 0.20 to 0.08)	0.612
*SATED questionnaire*	7.80 (7.50 to 8.10)	8.31 (8.00 to 8.62)	8.41 (8.11 to 8.70)	0.51 (0.13 to 0.89)	0.005	0.61 (0.25 to 0.97)	< 0.001	0.10 ( − 0.28 to 0.48)	0.804
*Epworth sleepiness scale (ESS)*	5.51 (5.04 to 5.97)	4.86 (4.37 to 5.35)	4.24 (3.77 to 4.71)	− 0.64 ( − 1.28 to 0.00)	0.049	− 1.27 ( − 1.88 to − 0.65)	< 0.001	− 0.62 ( − 1.26 to 0.02)	0.060

*CI*, confidence interval; *SATED*, satisfaction Alertness timing efficiency duration

The groups identified based on the PSQI score in the first visit (short-term) and the presence of a MCII in the PSQI score over the follow-up period showed few differences at baseline (Additional file 1: Table S2). The healthy group had a higher prevalence of males, while the MCII group exhibited a lower APACHE-II score. Differently, marked differences in sleep quality were evident at the short-term visit (Additional file 1: Table S3 and Figure S3). The median [p_25_;p_75_] PSQI scores were 2.93 [2.63; 3.22] in the healthy group, 9.08 [8.33; 9.83] in the non-MCII group, and 11.0 [9.82; 12.2] in the MCII group.

**Fig. 1:**
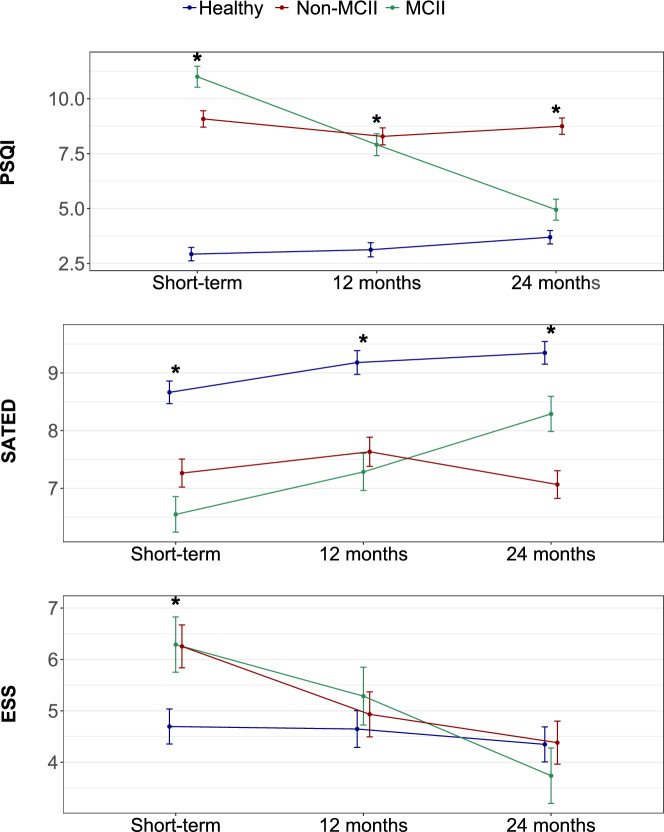
Trajectories of sleep. Data are represented as least square means (± SEM). The p-value threshold defining statistical significance was set at less than 0.05. ESS, Epworth Sleepiness Scale; MCII, minimal clinically important improvement; PSQI, Pittsburgh Sleep Quality Index; SATED, Satisfaction Alertness Timing Efficiency Duration; SEM, standard error of the mean

Over the follow-up period, PSQI scores remained stable for both the healthy and non-MCII groups (Fig. 1 and Additional file 1: Figure S4). The healthy group consistently maintained scores within the normal range (PSQI ≤ 5), while the non-MCII group remained in the poor sleep quality range (PSQI > 5). In contrast, the MCII group showed a quasi-linear improvement in sleep quality, with scores gradually aligning with those of the healthy group by the 24-month follow-up.

These PSQI trajectories were partially supported by the patterns observed in the SATED questionnaire. However, the ESS trajectories followed a distinct pattern: the ESS score of the healthy group remained stable throughout the follow-up period, while both the MCII and non-MCII groups demonstrated declines in ESS scores. By the 24-month follow-up, all groups achieved ESS scores comparable to those of the healthy group.

### Trajectories of other sequelae based on the observed sleep trajectories

Given the close association between sleep and other relevant factors for the recovery of critical survivors, we performed an additional analysis to investigate whether the trajectories of other sequelae were related to the observed sleep trajectories (as measured by the PSQI).

**Fig. 2:**
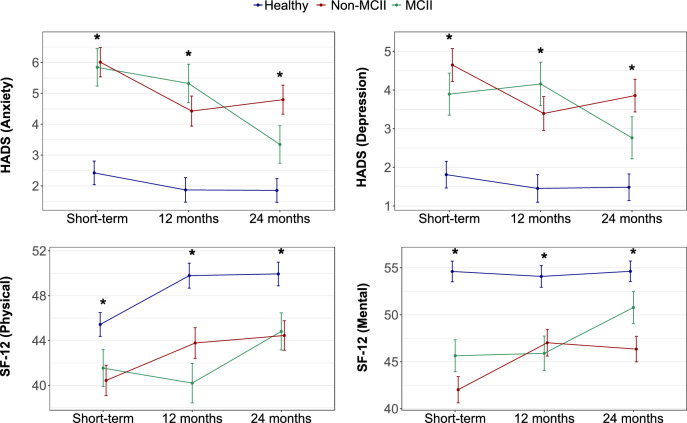
Trajectories of other sequelae based on the observed sleep trajectories. Data are represented as least square means (± SEM). The p-value threshold defining statistical significance was set at less than 0.05. HADS, Hospital Anxiety and Depression Scale; MCII, minimal clinically important improvement; SEM, standard error of the mean; SF-12, 12-item Short Form Survey

The trajectories of the HADS anxiety score, HADS depression score, and SF-12 mental score closely mirrored that of the PSQI score (Fig. 2 and Additional file 1: Figure S5). Specifically, the MCII group showed progressive improvement in these scores over time, ultimately reaching values closer to those of the healthy group by the 24-month follow-up. In contrast, the non-MCII group exhibited no significant changes. Notably, the trajectories of PP-6MWD, SpO_2_ in the 6MWT, DLCO, and total severity score were not related to the PSQI score trajectories (Additional file 1: Figure S6).

## Discussion

Our comprehensive analysis revealed distinct trajectories of sleep over the 24 months following the acute phase of critical illness, primarily defined by short-term sleep quality and the degree of improvement over time. Specifically, one group of patients exhibited good sleep quality in the short-term and maintained it throughout the 24 months. In contrast, other groups demonstrated poor sleep quality at this initial time-point. Among these, the group classified as MCII showed progressive improvement, ultimately achieving sleep quality levels comparable to the healthy group by the 24-month follow-up. Conversely, the group classified as non-MCII consistently exhibited poor sleep quality throughout the entire period. Further analyses revealed that these sleep trajectories were closely aligned with those of mental health.

Prior studies indicate a high prevalence of sleep alterations or poor sleep quality among critical patients during and after hospitalization [12–14]. We previously demonstrated that up to 60.5% of critical COVID-19 patients experienced poor sleep quality three months post-hospital discharge [17], with a slight improvement observed at mid-term (6 months after hospital discharge) [18] and long-term (12 months after hospital discharge) evaluations [19], yet still worse compared to the general population[37]. Despite the persistence of poor sleep quality in a considerable proportion of patients at the 12-month follow-up, studies with assessments exceeding this period are limited. To our knowledge, this is the first study to comprehensively evaluate this, revealing that while a subset of patients with initially poor sleep quality eventually achieves levels comparable to those with consistently good sleep, a significant proportion of individuals does not show a clinically relevant improvement in sleep quality during the follow-up period.

Several factors may influence the sleep of critical patients. The ICU environment is associated with lack of natural light exposure, inappropriate timing of artificial light exposure, nighttime care interventions, and noise from alarms and staff conversations. Critical patients often experience symptoms of pain and increased inflammatory processes, being under the effects of sleep-altering medications [38, 39]. Despite exposure to the same context, distinct sleep trajectories were observed, primarily defined by short-term sleep quality and the degree of improvement over time. We observed a higher prevalence of males among those with good short-term sleep quality, supporting our previous study, which identified female sex as a predictor of poorer sleep quality among critical COVID-19 patients at the 3-month follow-up [17]. This suggests that sex may be an important factor influencing sleep quality during the first few months after hospital discharge. In contrast, no baseline differences that could account for the divergences in sleep trajectories between the MCII and non-MCII groups were found. The absence of differences in hospitalization duration and ICU length of stay suggests that factors other than disease severity contribute to the MCII in sleep in this context. Notably, we observed a strong association between the MCII in sleep and factors such as anxiety, depression, and the mental domain of quality of life throughout the 24-month follow-up.

The observed relationship between sleep and mental health is supported by numerous studies. Consistent with this, we previously demonstrated positive correlations between these factors at three [17], six [18], and 12 months [19] post-hospital discharge among critical COVID-19 survivors. Our current findings reinforce this intimate connection, suggesting that the restoration of sleep quality two years after critical illness is associated with an improvement of mental health. Although the design of the current study precludes definitive conclusions regarding causality, it highlights sleep as a crucial component, alongside mental health, within the framework of post-intensive care syndrome. Importantly, the trajectories of sleep and respiratory function-related factors appear to be somewhat independent, suggesting distinct underlying mechanisms.

The current data should be interpreted in light of some aspects. First, although 332 patients were recruited during their ICU stay, 196 completed the 24-month follow-up and were therefore included in the analysis. Patients who were not included were relatively younger, spent fewer days in the hospital and at the ICU (Additional file 1: Table S1). This suggests that those who experienced more severe disease during the acute phase were more likely to complete the 24-month follow-up. Thus, the generalization of the findings presented here should be made with caution. Second, our cohort exclusively comprised critical survivors of SARS-CoV-2 infection. While there may be similarities in sleep-related outcomes among different populations of critical patients [16], subtle differences may exist depending on the cause of ICU admission. Yet, the evaluation of a well-characterized and highly homogeneous cohort minimizes the impact of condition-associated confounders. Third, the patients were recruited during the ICU stay to take advantage of the unprecedented natural research opportunity provided by the SARS-CoV-2 pandemic. While previous sleep disorders were investigated through available medical records, no prior assessments of sleep quality were conducted. The lack of improvement in the non-MCII group could, in fact, be attributed to an overlooked preexisting condition. However, this group did show improvement in daytime sleepiness, as indicated by the ESS score. Additionally, the recovery trajectory of the MCII group, which ultimately achieved sleep quality comparable to the healthy group, suggests that the decreased sleep quality reported by these patients shortly after the acute phase of critical illness is more likely a consequence of the disease and/or the ICU environment, rather than a preexisting condition. Fourth, given the observational design of the study, causal relationships between the correlated sequelae should be interpreted carefully. While numerous studies demonstrate a close relationship between sleep and mental health, the precise directionality, if any, cannot be confirmed with the current data.

## Conclusions

Our findings reveal that a subset of critical illness survivors requires up to 24 months after the acute phase to fully restore their sleep quality, while a significant proportion does not experience a clinically significant improvement in sleep quality over this period. These distinct sleep trajectories are strongly correlated with mental health status, highlighting the importance of addressing sleep alongside mental health within the framework of post-intensive care syndrome. Such an approach has the potential to improve patients' quality of life and promote a comprehensive recovery.

## Supplementary Information

Additional file 1

## Data Availability

The datasets used and/or analyzed during the current study are available from the corresponding author on reasonable request.

## Abbreviations

6MWD: 6-Minute walked distance
6MWT: 6-Minute walking test
BMI: Body mass index
COVID-19: Coronavirus disease 2019
DLCO: Diffusing lung capacity for carbon monoxide
ESS: Epworth sleepiness scale
HADS: Hospital anxiety and depression scale
ICU: Intensive care unit
IMV: Invasive mechanical ventilation
MCII: Minimal clinically important improvement
NIMV: Noninvasive mechanical ventilation
PP-6MWD: Percent predicted 6MWD
PSQI: Pittsburgh sleep quality index
SARS-CoV-2: Severe acute respiratory syndrome coronavirus 2
SATED: Satisfaction alertness timing efficiency duration
SF-12: 12-Item short form survey
