# Supplementary material for: The trajectory of sleep after critical illness: a 24-month follow-up study
Source: Ann Intensive Care. 2025 Feb 28;15:28. doi: 10.1186/s13613-025-01449-9 (PMC11871202; doi:10.1186/s13613-025-01449-9)
Supplement: Supplementary file 1 — Additional file 1 [file 13613_2025_1449_MOESM1_ESM.docx]

**The trajectory of sleep after critical illness: a 24-month follow-up study**

Mario Henríquez-Beltrán^1,2,3*^, Ms.C.; Iván D. Benítez^1,2*^, Ph.D.; Rafaela Vaca^1,2^, Ms.C.; Sally Santisteve^1^, Ms.C.; Maria Aguilà^1^, Ms.C.; Anna Vila^1^, Ms.C.; Olga Minguez^1,2^, Ms.C.; Carlos Rodríguez-Muñoz^1,2^, Ms.C.; Anna Galan Gonzalez^1^, Ms.C.; Sulamita Carvalho-Brugger^4^, M.D.; Paula González^5^, M.D.; Paula Rodríguez^4^, M.D.; Jesús Caballero^4^, M.D.; Carme Barberà^5^, M.D.; Gerard Torres^1,2^, M.D.; Gonzalo Labarca^6,7^, M.D.; Mar Malla-Banyeres^1^, Ms.C.; Anna Moncusí-Moix^1,2^, Ms.C.; Antoni Torres^2,8^, M.D.; David de Gonzalo-Calvo^1,2^, Ph.D.; Ferran Barbé^1,2^, M.D.; Jessica González^1,2^, M.D.; Adriano D. S. Targa^1,2#^, Ph.D.

^1^ Translational Research in Respiratory Medicine, Hospital Universitari Arnau de Vilanova-Santa Maria, Biomedical

Research Institute of Lleida (IRBLleida), Lleida, Spain.

^2^ CIBER of Respiratory diseases (CIBERES), Institute of Health Carlos III, Madrid, Spain.

^3^ Núcleo de Investigación en Ciencias de la Salud, Universidad Adventista de Chile, Chillán, Chile.

^4^ Intensive Care Department, Hospital Universitari Arnau de Vilanova, Lleida, Spain.

^5^ Intensive Care Department, Hospital Universitari Santa Maria, Lleida, Spain.

^6^ Department of Respiratory Diseases, School of Medicine, Pontificia Universidad Católica de Chile, Santiago, Chile.

^7^ Division of Pulmonary and Critical Care Medicine, Beth Israel Deaconess Medical Center and Harvard Medical School, Boston, United States.

^8^ Servei de Pneumologia, Hospital Clinic, Universitat de Barcelona, IDIBAPS, Barcelona, Spain.

*****Co-first authors. MHB and IB contributed equally to this study.

**^#^Corresponding author:**

Adriano D. S. Targa (atarga@irblleida.cat)

Biomedical Research Institute of Lleida (IRBLleida), Rovira Roure, 80, 25198, Lleida, Spain.

**eFigure 1.** Trajectory of sleep along the 24-month follow-up.

**eFigure 2.** Trajectory of sleep quality subdomains (PSQI) along the 24-month follow-up.

**eFigure 3.** Radar plot illustrating the scores for each component of the PSQI at the short-term for each study group.

**eFigure 4.** Trajectory of sleep quality along the 24-month follow-up.

**eFigure 5.** Radar plot illustrating the mean values for each marker of the distinct sequelae at the short-term for each study group.

**eFigure 6.** Trajectories of other sequelae based on the observed sleep trajectories.

**eTable 1.** Baseline characteristics of the included and non-included patients.

**eTable 2.** Baseline characteristics of the cohort.

**eTable 3.** Sleep quality at the short-term.

**eFigure 1.** Trajectory of sleep along the 24-month follow-up.


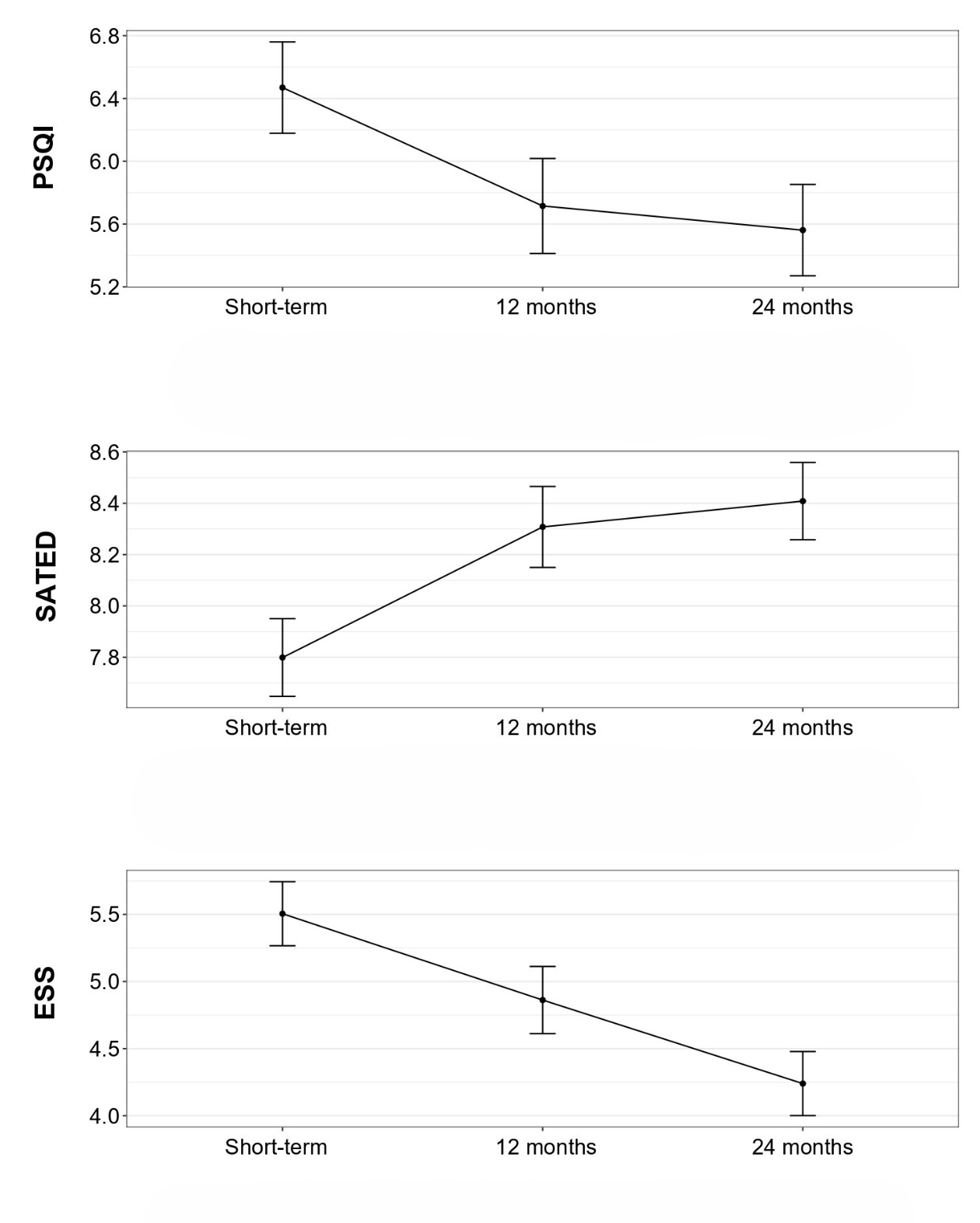


Data are represented as least square means (±SEM). The p-value threshold defining statistical significance was set at less than 0.05. ESS, Epworth Sleepiness Scale; PSQI, Pittsburgh Sleep Quality Index; SATED, Satisfaction Alertness Timing Efficiency Duration; SEM, standard error of the mean.

**eFigure 2.** Trajectory of sleep quality subdomains (PSQI) along the 24-month follow-up.


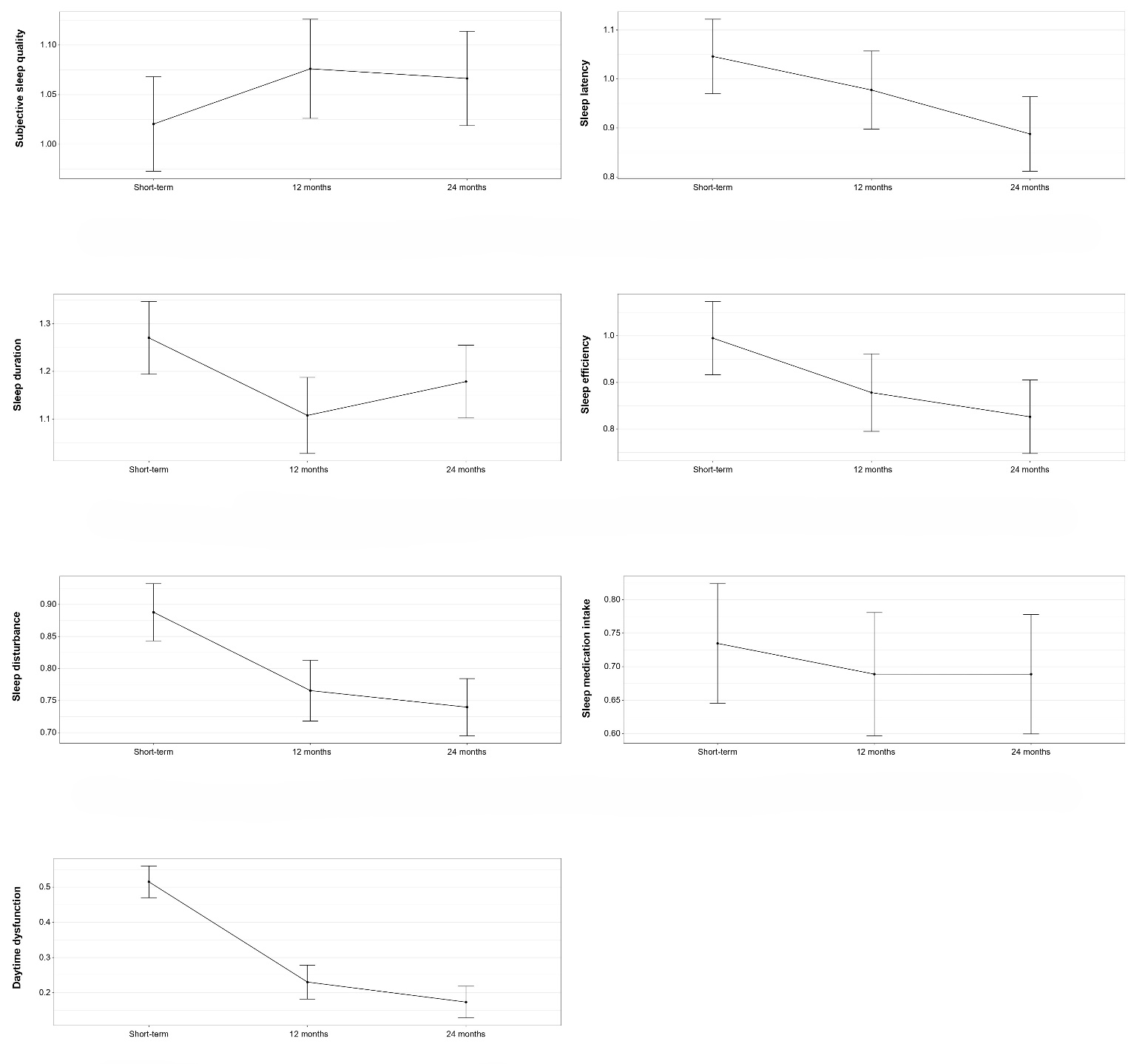


Data are represented as least square means (±SEM). The p-value threshold defining statistical significance was set at less than 0.05. PSQI, Pittsburgh Sleep Quality Index; SEM, standard error of the mean.

**eFigure 3.** Radar plot illustrating the scores for each component of the PSQI at the short-term for each study group.


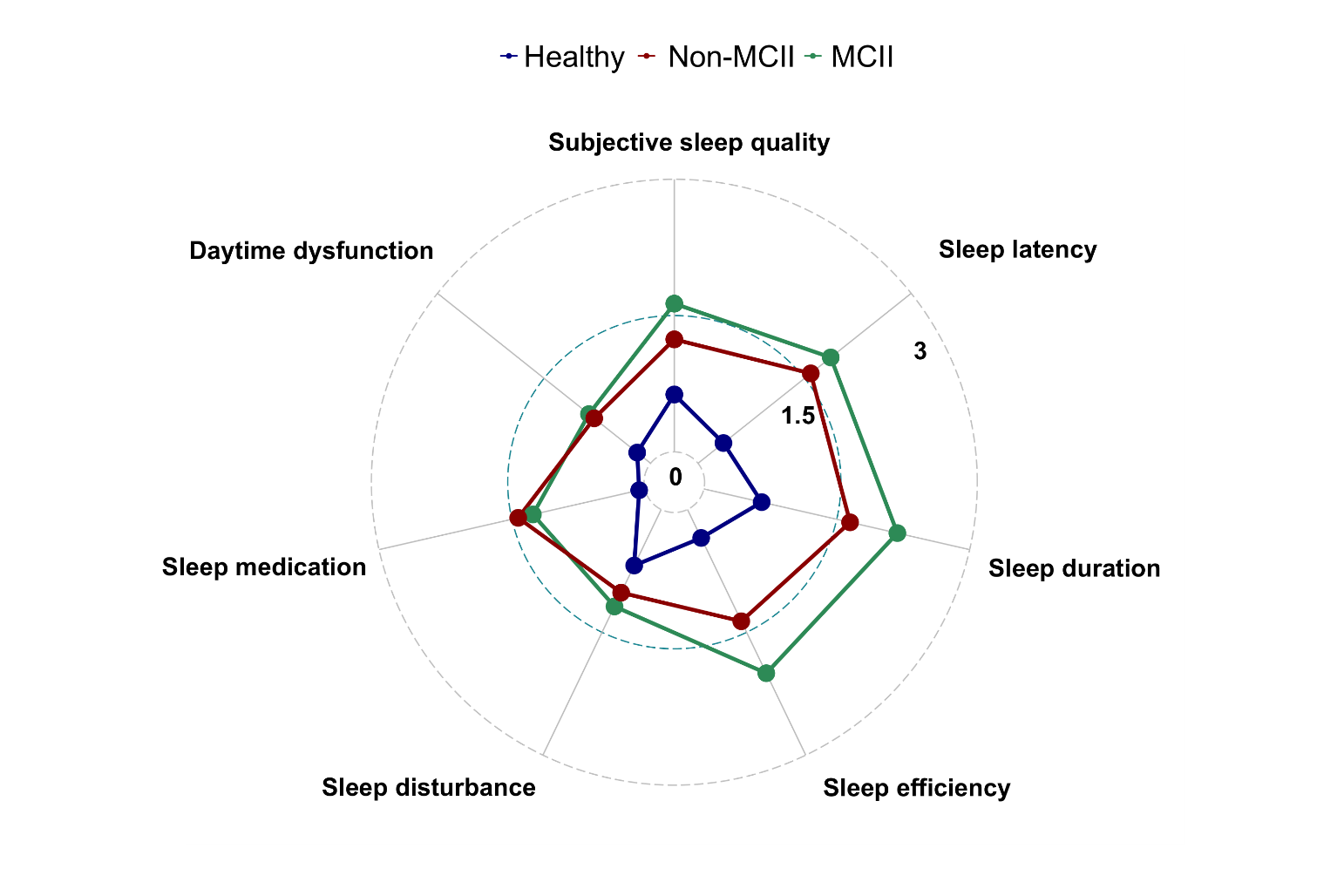


MCII, minimal clinically important improvement. PSQI, Pittsburgh Sleep Quality Index.

**eFigure 4.** Trajectory of sleep quality along the 24-month follow-up.


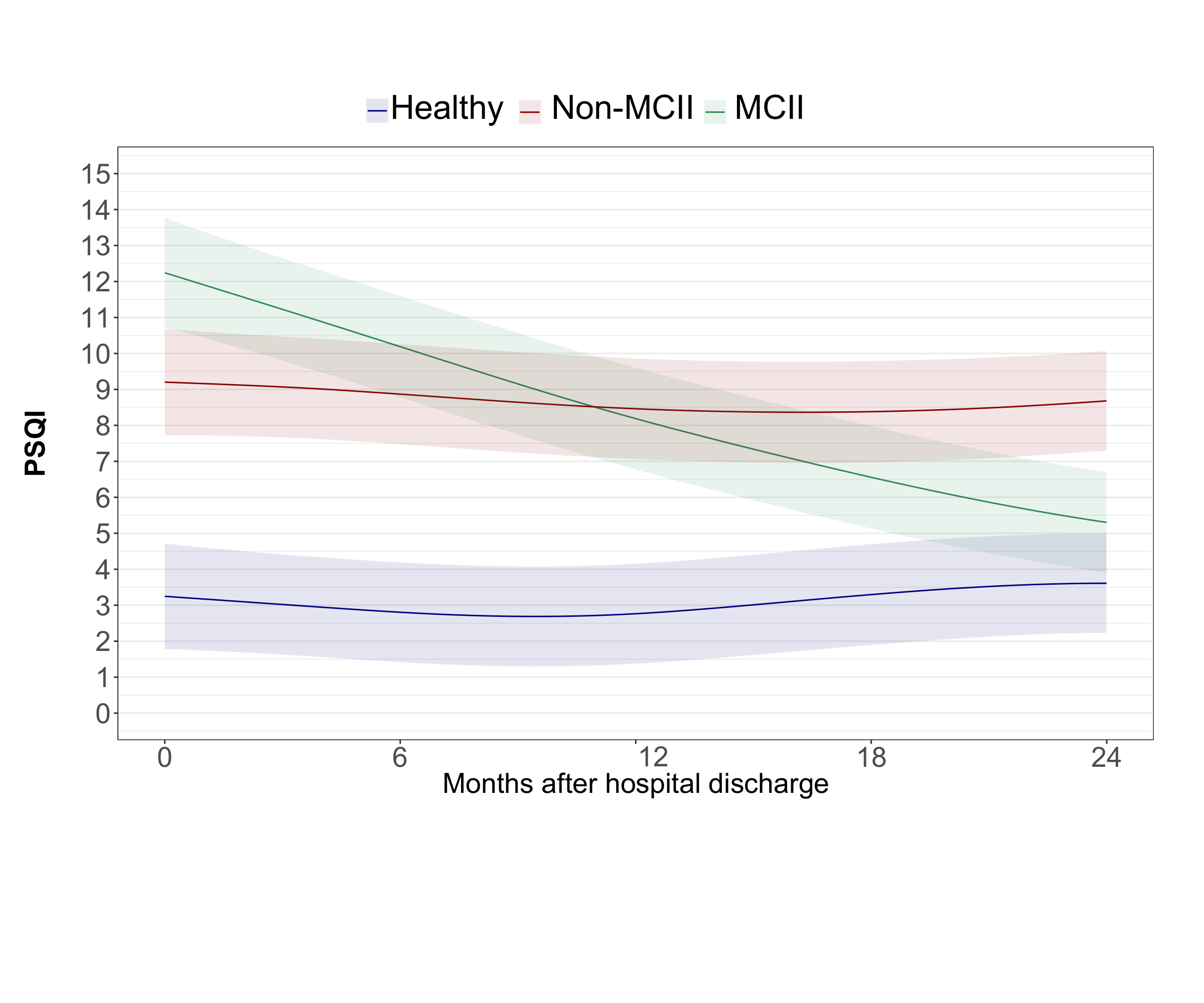


MCII, minimal clinically important improvement; PSQI, Pittsburgh Sleep Quality Index.

**eFigure 5.** Radar plot illustrating the mean values for each marker of the distinct sequelae at the short-term for each study group.


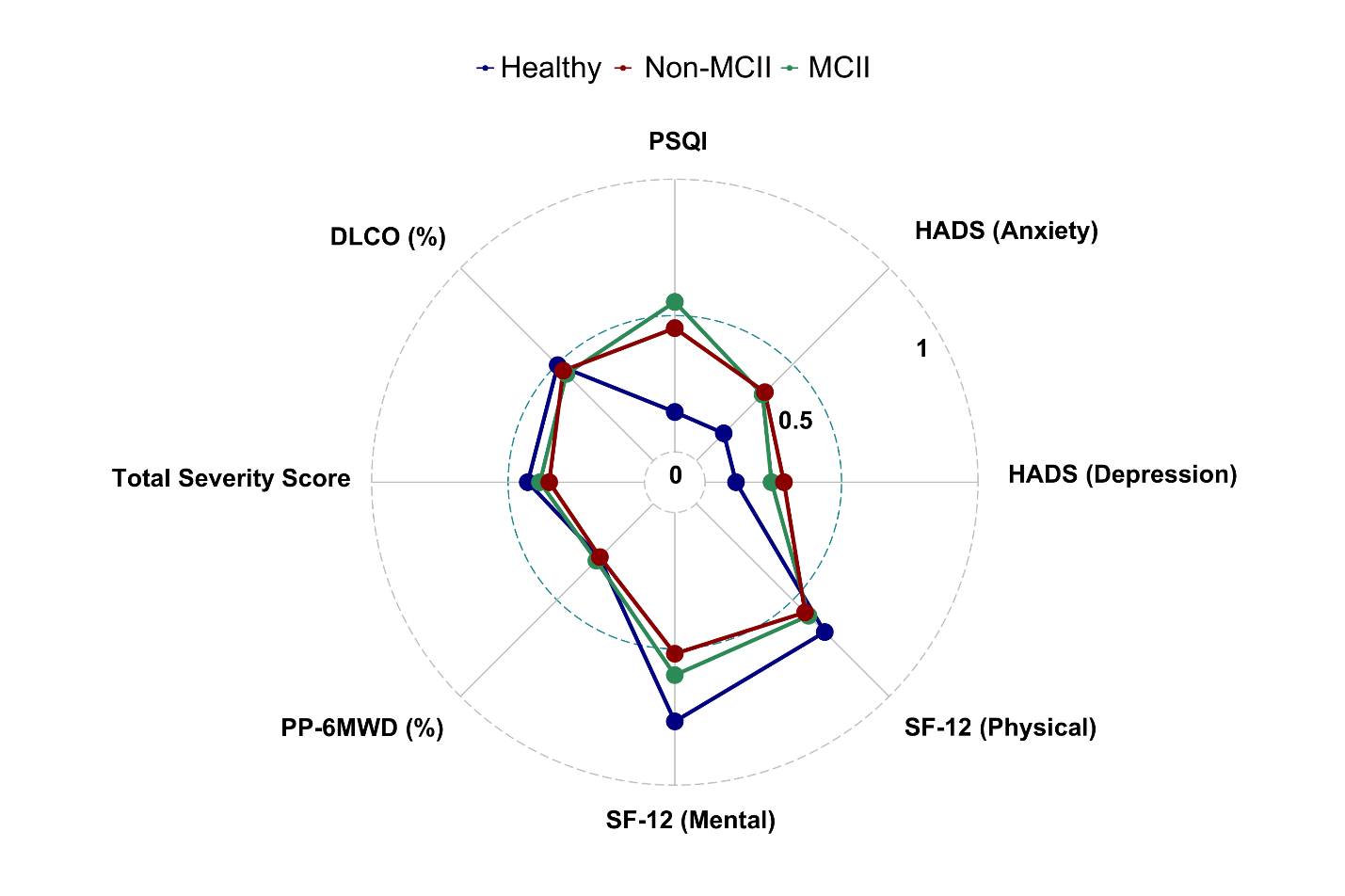


Variables were homogenized rescaling values from 0 to 1. 6MWD, 6-minute walked distance; DLCO, diffusing lung capacity for carbon monoxide; HADS, Hospital Anxiety and Depression Scale; MCII, minimal clinically important improvement; PP-6MWD, percent predicted 6MWD; PSQI, Pittsburgh Sleep Quality Index; SF-12, 12-item Short Form Survey.

**eFigure 6.** Trajectories of other sequelae based on the observed sleep trajectories.


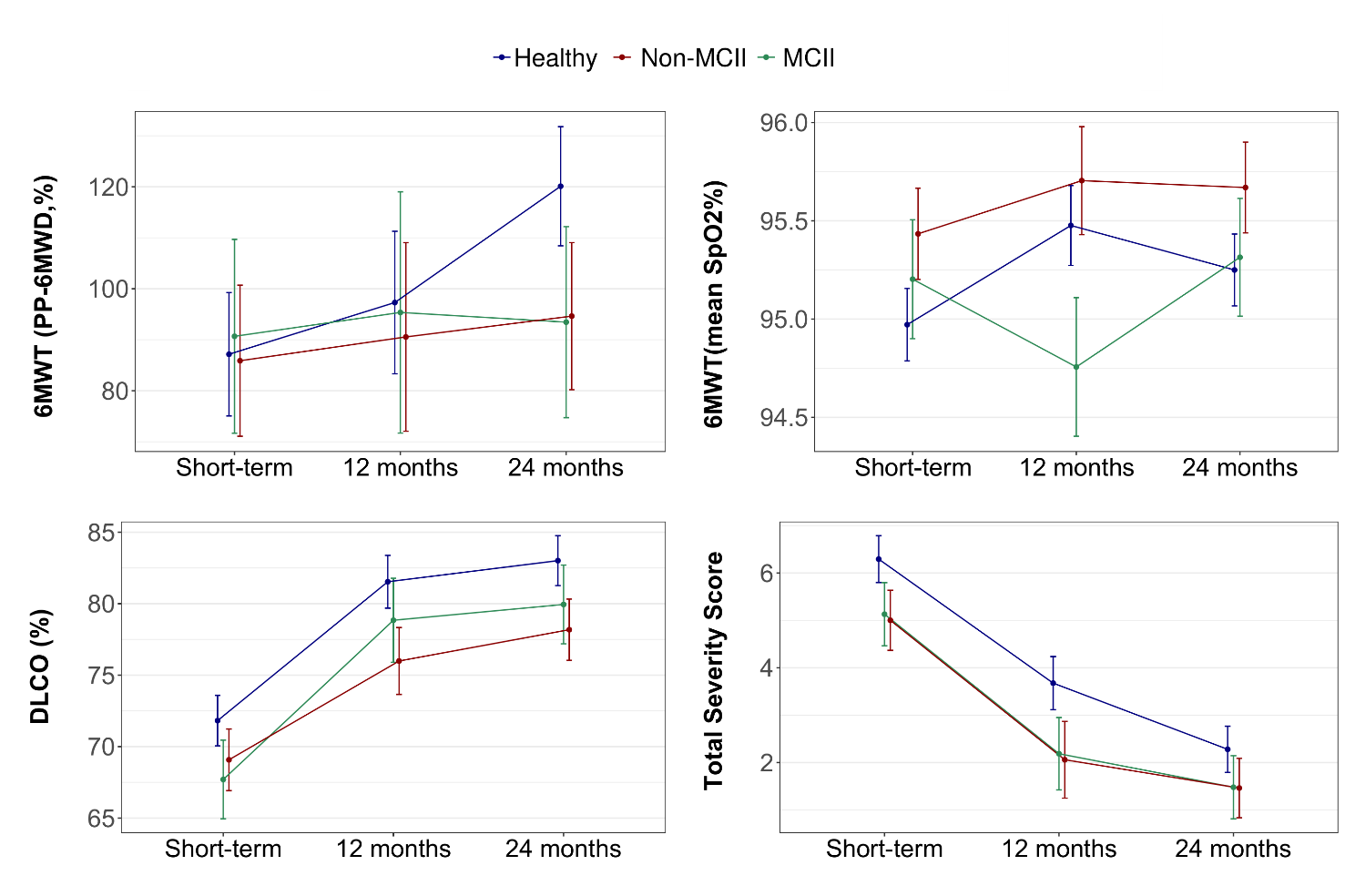


Data are represented as least square means (±SEM). The p-value threshold defining statistical significance was set at less than 0.05. 6MWD, 6-minute walked distance; 6MWT, 6-minute walking test; DLCO, diffusing lung capacity for carbon monoxide; MCII, minimal clinically important improvement; PP-6MWD, percent predicted 6MWD; SEM, standard error of the mean.

**eTable 1.** Baseline characteristics of the included and non-included patients.

|  | | **All** | **Included** | **Not included** | *p-value* |
| --- | --- | --- | --- | --- | --- |
|  | | n=332 | n=196 | n=136 |  |
|  | | *n (%), mean (SD) or median [p25;p75]* | | | |
| **Sociodemographic and anthropometric data** | |  |  |  |  |
| Sex, male | | 222 (66.9%) | 137 (69.9%) | 85 (62.5%) | 0.197 |
| Age, years | | 61.0 [51.0;67.0] | 62.0 [53.0;67.2] | 59.0 [49.0;66.0] | 0.030 |
| Body mass index, kg·m^2^ | | 30.1 [26.7;34.5] | 29.8 [26.7;33.6] | 30.5 [27.0;35.2] | 0.153 |
| **Habits** | |  |  |  |  |
| Tobacco | |  |  |  | 0.685 |
| *Former use* | | 153 (47.8%) | 91 (48.4%) | 62 (47.0%) |  |
| *Never used* | | 156 (48.8%) | 92 (48.9%) | 64 (48.5%) |  |
| *Current use* | | 11 (3.44%) | 5 (2.66%) | 6 (4.55%) |  |
| Chronic alcohol | |  |  |  | 0.500 |
| *Former consumption* | | 5 (1.55%) | 2 (1.06%) | 3 (2.26%) |  |
| *Never consumed* | | 303 (94.1%) | 180 (95.2%) | 123 (92.5%) |  |
| *Current consumption* | | 14 (4.35%) | 7 (3.70%) | 7 (5.26%) |  |
| **Comorbidities** | |  |  |  |  |
| Obesity | | 169 (50.9%) | 93 (47.4%) | 76 (55.9%) | 0.162 |
| Hypertension | | 147 (44.3%) | 90 (45.9%) | 57 (41.9%) | 0.542 |
| Diabetes mellitus type 2 | | 73 (22.0%) | 43 (21.9%) | 30 (22.1%) | 1.000 |
| Asthma | | 18 (5.42%) | 13 (6.63%) | 5 (3.68%) | 0.356 |
| Chronic obstructive pulmonary disease | | 15 (4.52%) | 8 (4.08%) | 7 (5.15%) | 0.849 |
| **Hospitalization** | |  |  |  |  |
| Duration, days | | 19.0 [13.0;32.0] | 22.0 [14.0;36.2] | 15.0 [11.0;26.0] | <0.001 |
| Before ICU admission, days | | 1.00 [0.00;2.00] | 1.00 [0.00;2.00] | 1.00 [0.00;2.00] | 0.792 |
| After ICU discharge, days | | 7.00 [4.00;10.2] | 8.00 [5.00;12.0] | 6.00 [4.00;9.00] | <0.001 |
| **ICU stay** | |  |  |  |  |
| Duration, days | | 10.0 [6.00;19.0] | 13.0 [7.00;22.0] | 8.00 [4.00;15.0] | <0.001 |
| APACHE-II score | | 10.0 [8.00;13.0] | 11.0 [9.00;13.0] | 9.00 [7.00;11.5] | 0.040 |
| ***Arterial blood-related information*** | |  |  |  |  |
| pH | | 7.45 [7.41;7.48] | 7.45 [7.40;7.48] | 7.45 [7.41;7.47] | 0.985 |
| Partial pressure of oxygen (PaO_2_), mmHg | | 69.0 [55.0;91.5] | 70.0 [56.0;91.0] | 65.0 [54.0;93.0] | 0.513 |
| Partial pressure of carbon dioxide (PaCO_2_), mmHg | | 36.0 [32.0;40.5] | 36.0 [32.2;41.0] | 35.0 [31.0;40.0] | 0.490 |
| Fractional inspired oxygen (FiO_2_), % | | 70.0 [50.0;85.0] | 65.0 [50.0;84.5] | 70.0 [50.0;85.8] | 0.731 |
| PaO_2_ to FiO_2_ ratio | | 126 [86.6;181] | 129 [90.1;172] | 104 [83.0;210] | 0.576 |
| Oxygen saturation (SaO_2_), % | | 95.0 [92.0;96.9] | 95.0 [91.3;96.9] | 94.8 [92.0;96.7] | 0.735 |
| Hydrogen carbonate (HCO_3_^-^), mmol/L | | 24.7 (3.79) | 24.6 (3.61) | 24.9 (4.12) | 0.559 |
| ***Procedures*** | |  |  |  |  |
| Invasive mechanical ventilation | | 170 (51.2%) | 108 (55.1%) | 62 (45.6%) | 0.111 |
| *Duration, days* | | 13.0 [7.00;25.0] | 13.0 [8.00;25.0] | 10.0 [6.00;24.0] | 0.097 |
| Non-invasive mechanical ventilation | | 264 (79.8%) | 147 (75.4%) | 117 (86.0%) | 0.026 |
| *Duration, days* | | 3.00 [1.25;5.00] | 3.00 [1.00;5.00] | 3.00 [2.00;6.00] | 0.559 |
| Prone position | | 166 (50.3%) | 111 (56.9%) | 55 (40.7%) | 0.005 |
| *Duration, hours* | | 37.5 [22.0;72.2] | 41.0 [24.0;78.8] | 27.5 [14.2;47.0] | 0.020 |
| ***Pharmacotherapy*** | |  |  |  |  |
| Corticosteroids | | 309 (93.4%) | 180 (91.8%) | 129 (95.6%) | 0.267 |
| Antibiotics | | 244 (73.5%) | 157 (80.1%) | 87 (64.0%) | 0.002 |
| Tocilizumab | | 241 (72.6%) | 135 (68.9%) | 106 (77.9%) | 0.090 |
| Hydroxychloroquine | | 62 (18.7%) | 48 (24.5%) | 14 (10.3%) | 0.002 |
| Remdesivir | | 35 (10.5%) | 25 (12.8%) | 10 (7.35%) | 0.163 |
|  |  |  |  |  |  |

APACHE-II, acute physiology and chronic health evaluation; FiO2, fractional inspired oxygen; ICU, intensive care unit; n, number; p, percentile; PaO2, partial pressure of oxygen; SD, standard deviation. Missings: tobacco, 12; chronic alcohol, 10.

**eTable 2.** Baseline characteristics of the cohort.

|  | **Healthy** | **Non-MCII** | **MCII** | *p-value* |
| --- | --- | --- | --- | --- |
|  | n=95 | n=63 | n=38 |  |
|  | *n (%) or median [p25;p75]* | | |  |
| **Sociodemographic and anthropometric data** |  |  |  |  |
| Sex, male | 76 (80.0%) | 38 (60.3%) | 23 (60.5%) | 0.011 |
| Age, years | 62.0 [54.5;69.5] | 61.0 [53.5;66.0] | 60.0 [50.0;65.0] | 0.191 |
| Body mass index, kg·m^2^ | 29.4 [26.7;34.3] | 31.2 [27.7;34.0] | 28.2 [25.1;30.8] | 0.095 |
| **Habits** |  |  |  |  |
| Tobacco |  |  |  | 0.369 |
| *Former use* | 48 (52.7%) | 29 (48.3%) | 14 (37.8%) |  |
| *Never used* | 42 (46.2%) | 29 (48.3%) | 21 (56.8%) |  |
| *Current use* | 1 (1.10%) | 2 (3.33%) | 2 (5.41%) |  |
| Chronic alcohol |  |  |  | 0.366 |
| *Former consumption* | 0 (0.00%) | 1 (1.61%) | 1 (2.78%) |  |
| *Never consumed* | 86 (94.5%) | 60 (96.8%) | 34 (94.4%) |  |
| *Current consumption* | 5 (5.49%) | 1 (1.61%) | 1 (2.78%) |  |
| **Comorbidities** |  |  |  |  |
| Obesity | 41 (43.2%) | 37 (58.7%) | 15 (39.5%) | 0.087 |
| Hypertension | 51 (53.7%) | 20 (31.7%) | 19 (50.0%) | 0.022 |
| Diabetes mellitus type 2 | 22 (23.2%) | 12 (19.0%) | 9 (23.7%) | 0.796 |
| Asthma | 4 (4.21%) | 3 (4.76%) | 6 (15.8%) | 0.048 |
| Chronic obstructive pulmonary disease | 5 (5.26%) | 1 (1.59%) | 2 (5.26%) | 0.562 |
| **Hospitalization** |  |  |  |  |
| Duration, days | 22.0 [15.0;34.0] | 23.0 [15.5;33.0] | 20.0 [13.0;43.0] | 0.988 |
| Before ICU admission, days | 1.00 [0.00;3.00] | 1.00 [0.00;2.00] | 0.00 [0.00;2.00] | 0.596 |
| After ICU discharge, days | 8.00 [5.00;13.0] | 8.00 [5.00;11.0] | 6.00 [4.00;12.8] | 0.468 |
| **ICU stay** |  |  |  |  |
| Duration, days | 13.0 [7.00;20.0] | 13.0 [7.00;23.0] | 12.0 [6.25;30.0] | 0.987 |
| APACHE-II score | 12.0 [9.00;14.0] | 11.0 [9.25;12.0] | 9.00 [7.50;9.50] | 0.024 |
| ***Arterial blood-related information*** |  |  |  |  |
| pH | 7.44 [7.37;7.48] | 7.45 [7.41;7.48] | 7.45 [7.42;7.47] | 0.900 |
| Partial pressure of oxygen (PaO_2_), mmHg | 70.0 [54.5;90.0] | 68.5 [58.5;89.5] | 74.0 [64.2;95.5] | 0.527 |
| Partial pressure of carbon dioxide (PaCO_2_), mmHg | 36.5 [32.0;43.2] | 36.0 [32.2;39.0] | 36.0 [34.8;42.0] | 0.691 |
| Fractional inspired oxygen (FiO_2_), % | 75.0 [50.0;90.0] | 60.0 [50.0;80.0] | 60.0 [50.0;80.0] | 0.134 |
| PaO_2_ to FiO_2_ ratio | 118 [81.2;147] | 140 [97.1;190] | 134 [102;186] | 0.090 |
| Oxygen saturation (SaO_2_), % | 94.9 [91.0;96.6] | 95.0 [92.5;97.0] | 94.9 [91.0;97.0] | 0.850 |
| Hydrogen carbonate (HCO_3_^-^), mmol/L | 24.7 [21.9;27.4] | 24.9 [21.8;28.5] | 24.6 [22.6;27.0] | 0.470 |
| ***Procedures*** |  |  |  |  |
| Invasive mechanical ventilation | 54 (56.8%) | 31 (49.2%) | 23 (60.5%) | 0.484 |
| *Duration, days* | 11.5 [7.00;18.0] | 17.0 [9.50;25.5] | 17.0 [10.0;27.5] | 0.247 |
| Non-invasive mechanical ventilation | 71 (74.7%) | 48 (77.4%) | 28 (73.7%) | 0.896 |
| *Duration, days* | 3.00 [1.00;5.00] | 3.00 [2.00;5.00] | 2.00 [1.00;4.50] | 0.661 |
| Prone position | 58 (61.1%) | 32 (51.6%) | 21 (55.3%) | 0.493 |
| *Duration, hours* | 48.0 [23.0;78.0] | 38.0 [24.0;76.0] | 40.0 [24.5;116] | 0.832 |
| ***Pharmacotherapy*** |  |  |  |  |
| Corticosteroids | 88 (92.6%) | 57 (90.5%) | 35 (92.1%) | 0.940 |
| Antibiotics | 76 (80.0%) | 46 (73.0%) | 35 (92.1%) | 0.067 |
| Tocilizumab | 64 (67.4%) | 50 (79.4%) | 21 (55.3%) | 0.037 |
| Hydroxychloroquine | 23 (24.2%) | 14 (22.2%) | 11 (28.9%) | 0.745 |
| Remdesivir | 15 (15.8%) | 5 (7.94%) | 5 (13.2%) | 0.355 |
|  |  |  |  |  |
|  |  | | | |

APACHE-II, acute physiology and chronic health evaluation; FiO2, fractional inspired oxygen; ICU, intensive care unit; MCII, minimal clinically important improvement; n, number; p, percentile; PaO2, partial pressure of oxygen. Missings: tobacco, 8; chronic alcohol, 7.

**eTable 3.** Sleep quality at the short-term.

|  | **Healthy** | **Non-MCII** | **MCII** |  | **Healthy vs. Non-MCII** | **Healthy vs. MCII** | **MCII vs. Non-MCII** |
| --- | --- | --- | --- | --- | --- | --- | --- |
|  | n=95 | n=63 | n=38 |  |  |  |  |
|  | *median (p25;p75)* | *median (p25;p75)* | *median (p25;p75)* | *p-value* | *p-value* | *p-value* | *p-value* |
| **PSQI total score** | 2.93 [2.63;3.22] | 9.08 [8.33;9.83] | 11.0 [9.82;12.2] | <0.001 | <0.001 | <0.001 | 0.001 |
| Subjective sleep quality | 0.63 [0.53;0.73] | 1.24 [1.08;1.40] | 1.63 [1.37;1.89] | <0.001 | <0.001 | <0.001 | 0.005 |
| Sleep latency | 0.36 [0.25;0.47] | 1.59 [1.32;1.85] | 1.87 [1.53;2.20] | <0.001 | <0.001 | <0.001 | 0.229 |
| Sleep duration | 0.65 [0.49;0.82] | 1.65 [1.39;1.91] | 2.18 [1.87;2.50] | <0.001 | <0.001 | <0.001 | 0.013 |
| Sleep efficiency | 0.35 [0.23;0.47] | 1.37 [1.07;1.66] | 2.00 [1.64;2.36] | <0.001 | <0.001 | <0.001 | 0.002 |
| Sleep disturbance | 0.68 [0.58;0.79] | 1.02 [0.84;1.19] | 1.18 [0.97;1.40] | <0.001 | 0.002 | <0.001 | 0.359 |
| Sleep medication intake | 0.06 [-0.03;0.15] | 1.43 [1.05;1.80] | 1.26 [0.80;1.73] | <0.001 | <0.001 | <0.001 | 0.740 |
| Daytime dysfunction | 0.19 [0.10;0.28] | 0.79 [0.55;1.03] | 0.87 [0.58;1.16] | <0.001 | <0.001 | <0.001 | 0.872 |

MCII, minimal clinically important improvement; n, number; p, percentile; PSQI, Pittsburgh sleep quality index.
